# The age-standardized incidence, mortality, and case fatality rates of COVID-19 in 79 countries: a cross-sectional comparison and their correlations with associated factors

**DOI:** 10.4178/epih.e2021061

**Published:** 2021-09-08

**Authors:** Dongui Hong, Sohyae Lee, Yoon-Jung Choi, Sungji Moon, Yoonyoung Jang, Yoon-Min Cho, Hyojung Lee, Sukhong Min, Hyeree Park, Seokyung Hahn, Ji-Yeob Choi, Aesun Shin, Daehee Kang

**Affiliations:** 1 Department of Preventive Medicine, Seoul National University College of Medicine, Seoul, Korea; 2 Integrated Major in Innovative Medical Science, Seoul National University Graduate School, Seoul, Korea; 3 Environmental Health Center, Seoul National University College of Medicine, Seoul, Korea; 4 Institute of Health and Environment, Seoul National University, Seoul, Korea; 5 Department of Human Systems Medicine, Seoul National University College of Medicine, Seoul, Korea; 6 Department of Biomedical Sciences, Seoul National University Graduate School, Seoul, Korea

**Keywords:** COVID-19, Incidence, Mortality, Vaccines, Infections, Aged

## Abstract

**OBJECTIVES:**

During the coronavirus disease 2019 (COVID-19) pandemic, crude incidence and mortality rates have been widely reported; however, age-standardized rates are more suitable for comparisons. In this study, we estimated and compared the age-standardized incidence, mortality, and case fatality rates (CFRs) among countries and investigated the relationship between these rates and factors associated with healthcare resources: gross domestic product per capita, number of hospital beds per population, and number of doctors per population.

**METHODS:**

The incidence, mortality, and CFRs of 79 countries were age-standardized using the World Health Organization standard population. The rates for persons 60 years or older were also calculated. The relationships among the rates were analysed using trend lines and coefficients of determination (R^2^). Pearson correlation coefficients between the rates and the healthcare resource-related factors were calculated.

**RESULTS:**

The countries with the highest age-standardized incidence, mortality, and CFRs were Czechia (14,253 cases/100,000), Mexico (182 deaths/100,000), and Mexico (6.7%), respectively. The R^2^ between the incidence and mortality rates was 0.852 for all ages and 0.945 for those 60 years or older. The healthcare resources-related factors were associated positively with incidence rates and negatively with CFRs, with weaker correlations among the elderly.

**CONCLUSIONS:**

Compared to age-standardized rates, crude rates showed greater variation among countries. Medical resources may be important in preventing COVID-19-related deaths; however, considering the small variation in fatality among the elderly, preventive measures such as vaccination are more important, especially for the elderly population, to minimize the mortality rates.

## INTRODUCTION

After the first suspected case of coronavirus disease 2019 (COVID-19) was identified on November 17, 2019, in Hubei Province in the People’s Republic of China, COVID-19 spread rapidly across borders [1]. By March 11, 2020, there were more than 118,000 confirmed cases and 4,291 deaths in 114 countries, and as a result, COVID-19 was declared a pandemic by the World Health Organization (WHO) [2]. The WHO reported that as of April 6, 2021, there were 131,077,562 confirmed cases and 2,852,221 deaths worldwide, with the United States accounting for the highest number of cases and deaths (30,372,016 cases and 551,391 deaths) [3].

During the COVID-19 pandemic, information on the number of cases and deaths has been communicated through various media channels, including online dashboards. Various organizations, including the WHO, Johns Hopkins University (JHU), and Worldometer, have been reporting global COVID-19 statistics [3-5]. The WHO has been reporting the daily and cumulative confirmed cases and deaths in 235 countries or areas and the JHU dashboard has been reporting cumulative cases, active cases, incidence rates, case fatality ratios, and testing rates by country, region, and sovereignty.

The current statistics available are limited to crude rates, which fail to account for the differences in age distribution across countries. Most previous studies that reported age-standardized rates were confined to a few countries [6], although a recent study reported the age-standardized COVID-19 mortality rates of 51 countries [7]. To enhance the comparability of rates among countries in a more comprehensive manner, we aimed to estimate and compare age-standardized incidence, mortality, and case fatality rates (CFRs) among countries throughout the world. In addition, we further analysed the relationship between age-standardized incidence, mortality, and CFRs and factors related to healthcare resources, such as gross domestic product (GDP) per capita, number of hospital beds per population, and number of doctors per population.

## MATERIALS AND METHODS

### Selection of countries

To maximize the number of countries included in the analysis, we investigated the availability of publicly released COVID-19 data from all countries. Data sources included situation reports from official national COVID-19 websites, dashboards, raw data, and, if these were not available, news articles. We included countries that had provided up-to-date data regarding the age distribution of COVID-19 cases and/or deaths as of April 6, 2021, resulting in a total of 79 countries. Among these countries, 16 countries provided age distribution data only for cases, 6 countries provided age distribution data only for deaths, and 57 countries provided age distribution data for both cases and deaths. Collective data were not available for the United Kingdom. For the analysis, we unified the age groups for cases and deaths in England, Scotland, Wales, and Northern Ireland. The specific sources of data are summarized in Supplementary Material 1.

### Age distribution of cases and deaths

Age group classifications varied across countries: some countries grouped cases or deaths according to 5-year age groups, while others used 10-year age groups. We used the following rules to unify the structure of the data while maintaining data integrity. Exceptions to these rules are summarized in the Supplementary Material 2.

#### Rule 1

Age groups with an age range smaller than a 10-year range (i.e., 5-year age groups) were converted to 10-year age groups; however, age groups with a range greater than 10 years were not converted to 10-year age groups due to bias associated with the transformation. Uniformity was compromised to minimize bias. Ten-year age groups grouped starting from 0-4 years (5-14, 15-24…) and 0-9 years (10-19, 20-29…) were used without any further conversion. When age groups differed between cases and deaths, age-standardized incidence and mortality rates were calculated independently based on the original age structure for cases and deaths (Argentina, Belgium, Bermuda, Chile, Finland, Luxembourg, Mozambique, and Norway); however, for CFR calculations, which require uniform age distributions for cases and deaths, the age groups for cases were transformed to match the age groups for deaths. For countries that reported age data for cases/deaths, cases/deaths were grouped into 10-year age groups, of which 80 and above was the oldest.

#### Rule 2

To analyse the age-standardized rates for those aged 60 or older for countries that used age groups that included the age of 60 (i.e., 55-64 years), the number of cases/deaths was divided proportionally according to the population distribution of those aged less than 60 and those aged 60 and above (i.e., 55-59 and 60-64 years).

#### Rule 3

In some countries, the total numbers of cases and deaths did not equal the sum of all cases or deaths for each age group. Agestandardized rates were underestimated for countries with greater proportions of unidentified ages for cases and deaths. To minimize the degree of underestimation, cases or deaths of unidentified age were redistributed among all age groups according to the known proportion of cases or deaths in each age group.

### Population of each country and standard population

Population data were obtained from the United Nations Population Division’s ‘‘World Population Prospects: The 2019 Revision” [8]. Alternative sources of data were used for countries not included in the report. For example, Taiwan’s population was sourced from the National Statistics of Taiwan [9], the population of the Faroe Islands was sourced from the Demographic Statistics Database maintained by the United Nations Statistics Division [10], the population of the Isle of Man was sourced from the 2016 Isle of Man Census Report [11], and Bermuda’s population was sourced from the Department of Statistics of Government of Bermuda [12]. Incidence, mortality, and CFRs were standardized using the WHO world standard population distribution (2000-2025) [13]. Exceptions with regard to population structure are summarized in the Supplementary Material 2.

### Case fatality rate

The CFR is the proportion of the number of deaths from COVID-19 compared to the number of confirmed cases of COVID-19 [14]. The CFR was calculated by the following equation:


$$CFR(\%)=100\times \frac{The\;number\;of\;deaths\;from\;COVID-19}{The\;number\;of\;confirmed\;cases\;of\;COVID-19}(\%)$$

### Other variables

The 2019 GDP per capita data were obtained from the International Monetary Fund [15]. There were some exceptions: GDP data for Lebanon, Bermuda, the Isle of Man (2018), and the Faroe Islands (2018) were retrieved from the World Bank [16]. The Central Intelligence Agency (CIA) World Factbook [17] provided information on the number of hospital beds per population. There were some exceptions: data for the United States and Nigeria were obtained from the WHO [18], data for Palestine were obtained from Palestinian Central Bureau of Statistics [19], data for Bermuda and the Isle of Man were obtained from the World Bank [20] and data for Macao and Taiwan were obtained from the National Bureau of Statistics of China [21]. Data on the number of medical doctors per population were obtained from the WHO [22]. There were some exceptions: data for Hong Kong, Macau, and the Faroe Islands were retrieved from the CIA World Factbook [23], data for Palestine were obtained from Palestinian Central Bureau of Statistics [17], data for Bermuda and the Isle of Man were obtained from the World Bank [24] and data for Taiwan were retrieved from the Taiwan health and welfare report [25].

### Statistical analysis

To account for the differences in age structure across all countries when comparing the incidence, mortality, and CFRs, direct age standardization was performed as follows:


$$I_{s}=100,000\times \sum_{k=1}^{n}w_{k}\frac{c_{k}}{p_{k}}\;M_{s}=100,000\times \sum_{k=1}^{n}w_{k}\frac{d_{k}}{p_{k}}\;F_{s}=100\times \sum_{k=1}^{n}w_{k}\frac{d_{k}}{c_{k}}$$

where *I_s_, M_s_, F_s_* are the age-standardized incidence, mortality, and CFRs, respectively. *k*= 1, …, *n* represents each age group, *w_k_* is the proportion of the standard population in the *k*th age group, *c_k_* is the number of cases in the *k*th age group, *d_k_* is the number of deaths in the *k*th age group, and *p_k_* is the population of the *k*th age group. The same method was used to calculate the truncated rates for those aged 60 years or older.

All variables were log-transformed to achieve a normal distribution. Trend lines and coefficients of determination (R^2^) were obtained to estimate the relationships among (1) the age-standardized incidence and mortality rates (all ages), (2) truncated age-standardized incidence and mortality rates (age≥ 60), and (3) CFRs for all ages and CFRs for those 60 years or older. Pearson correlation coefficients between the standardized rates and the factors related to healthcare resources, such as GDP per capita, the number of hospital beds per population, and the number of doctors per population, were calculated. All analyses were performed with SAS version 9.4 (SAS Institute Inc., Cary, NC, USA), and statistical significance was set at the level of α= 0.05.

### Ethics statement

The study was approved as exempt by the Institutional Review Board of Seoul National University College of Medicine/Seoul National University Hospital (E-2012-110-1183) because all analyses were conducted using publicly available data without personal identification information.

## RESULTS

The crude and age-standardized incidence, mortality, and CFRs in 79 countries from 6 continents as of April 6, 2021 are presented in Table 1. Czechia, Montenegro, Luxembourg, Israel, and Slovenia showed the highest age-standardized incidence rates, with 14,253, 13,866, 10,145, 9,966, and 9,916 cases per 100,000, respectively. The top 5 countries with the highest age-standardized incidence rates differed for those aged 60 years or older; these age-standardized incidence rates were the highest in Montenegro, Czechia, Lebanon, Palestine, and Moldova, with 14,973, 12,778, 12,015, 11,367, and 9,689 cases per 100,000, respectively. The top 5 countries in terms of crude incidence rates were Montenegro, Czechia, Slovenia, Luxembourg, and Israel. For those aged 60 years or older, the top 5 countries in respect of crude incidence rates were the same as those for the age-standardized incidence rates for those 60 years or older.

The age-standardized mortality rate rankings differed from the age-standardized incidence rate rankings. The top 5 countries, in order, were Mexico, Peru, Panama, Palestine, and Jordan, with 182.1, 152.9, 134.2, 130.3, and 130.2 deaths per 100,000, respectively. For those aged 60 years or older, Mexico, Palestine, Peru, Jordan, and Panama showed the highest age-standardized mortality rates, with 992.4, 942.9, 879.2, 876.4, and 831.3 deaths per 100,000, respectively. The crude mortality rates for all ages were the highest in Czechia, Hungary, Bosnia and Herzegovina, Montenegro, and Belgium, whereas Mexico, Czechia, Peru, Panama, and Palestine showed the highest crude mortality rates for those aged 60 years or older. For all countries, the truncated age-standardized mortality rates (age≥ 60) were higher than the overall age-standardized mortality rates; however, there was no consistency with regard to the age-standardized incidence rates.

The age-standardized CFRs were highest in Mexico (6.7%), Eswatini (3.7%), Ecuador (3.5%), Guatemala (3.4%), and Indonesia (2.5%). For those aged 60 years or older, Mexico (34.1%), Eswatini (19.8%), Ecuador (19.0%), Fiji (18.6%), and Guatemala (17.4%) showed the highest age-standardized CFRs. The crude CFRs were highest in Mexico, Ecuador, Bosnia and Herzegovina, Bulgaria, and Eswatini. For those aged 60 years or older, Mexico, Fiji, Eswatini, Ecuador, and Guatemala showed the highest crude CFRs. For all countries, the truncated CFRs (age≥ 60) were higher than those for all ages.

The crude rates were found to have greater variation than the age-standardized rates. The standard deviations of age-standardized incidence, mortality, and CFRs were 3,474.7, 45.6, and 1.1, respectively. They were smaller than those for the crude rates (3,547.6, 73.7, and 1.4 for the crude incidence, mortality, and CFRs, respectively).

**Figure 1.**
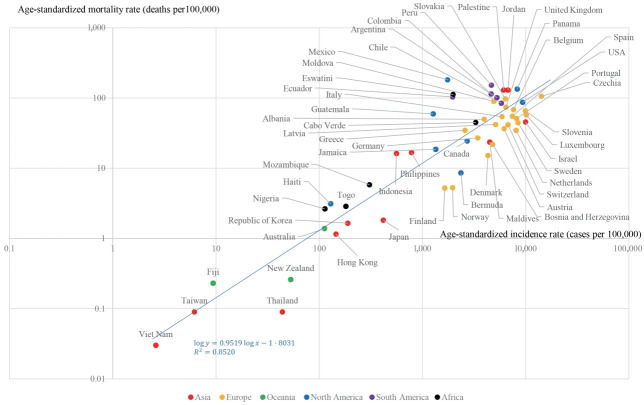
Age-standardized incidence and mortality rates (all ages). R^2^, coefficients of determination.

**Figure 2.**
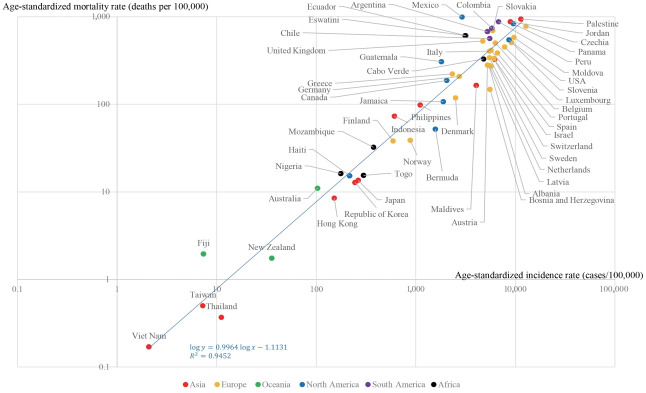
Age-standardized incidence and mortality rates (age≥60). R^2^, coefficients of determination.

**Figure 3.**
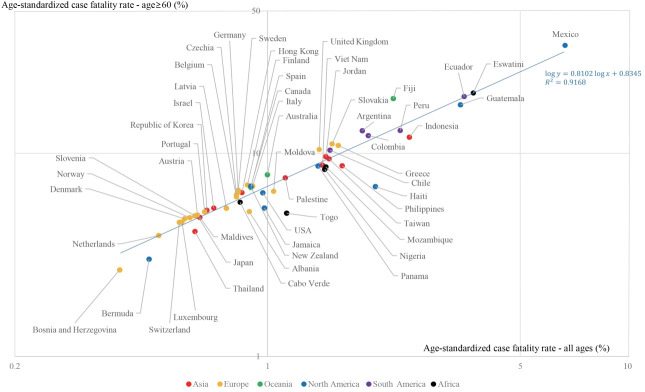
Age-standardized case fatality rates – all ages and age≥60. R^2^, coefficients of determination.

A strong correlation between age-standardized incidence and age-standardized mortality rates was found (Figure 1). The R^2^ between the log-transformed age-standardized incidence and log-transformed age-standardized mortality rates was 0.852. A stronger correlation (R^2^=0.945) was found between the truncated log-transformed age-standardized incidence (age≥ 60) and truncated log-transformed age-standardized mortality rates (age≥ 60) (Figure 2). The CFRs for all ages and those for individuals aged 60 years or older also showed a strong correlation (R^2^= 0.917) (Figure 3).

Table 2 shows the Pearson correlation coefficients among the log-transformed variables used in this study. The incidence rates showed a positive correlation with the number of doctors per population (r= 0.47, p< 0.001). With regard to the mortality rate, only the number of doctors per population showed a significantly positive correlation (r= 0.29, p= 0.023). However, the CFR showed negative correlation coefficients with GDP per capita (r= -0.54, p< 0.001), the number of beds per population (r= -0.56, p< 0.001), and the number of doctors per population (r= -0.37, p= 0.005). In analyses limited to the elderly, weaker correlations were shown. The incidence rate showed a positive correlation with the number of doctors per population (r= 0.37, p= 0.001). The number of doctors per population showed a positive correlation with the truncated age-standardized mortality rate (age≥ 60). However, the truncated CFR (age≥60) showed negative correlation coefficients with GDP per capita (r= -0.36, p= 0.006) and the number of beds per population (r= -0.41, p= 0.002).

## DISCUSSION

In this study, we calculated the age-standardized incidence, mortality, and CFRs for all ages and for those aged 60 years or older. The incidence and mortality rates showed a strong correlation. CFRs were negatively correlated with GDP per capita, the number of hospital beds per population, and the number of doctors per population.

This is the first study to compare age-standardized incidence rates and CFRs among the largest possible number of countries worldwide; however, a recent study compared age-standardized COVID-19 mortality rates among 51 countries [7]. In addition, we also compared standardized rates for those aged 60 years or older and analysed the correlations between the standardized rates and factors associated with medical resources, namely, GDP per capita, the number of hospital beds per population, and the number of doctors per population.

Age adjustment is important when comparing incidence, mortality, and fatality rates among countries due to differences in age structures. Interpreting disease patterns across countries using non-standardized rates could lead to significant bias because differences between crude rates may be due to differences in age structures. A study regarding the comparison of age-standardized mortality rates of COVID-19 also argued that the age-standardization is necessary for comparing mortality rates across countries [7]. For example, Czechia was ranked first for the crude mortality rate, but ninth for the age-standardized mortality rate. This could have been because these countries have older populations, leading to higher crude mortality rates.

The value of age-standardized rates could be different according to the source of the population structure of each country, the standard population used for age-standardization, the intervals of the age groups, and when the data regarding COVID-19 were collected. The calculated age-standardized mortality rates reported in a recent study were similar to those in our study because the dates of data collection were close (both were collected in April 2021), an identical standard population was used, and both studies sourced their population structure from the United Nations [7].

Variations in incidence, mortality, and CFRs may be attributable to differences in test rates, methods for confirming deaths due to COVID-19, and detection rates of asymptomatic patients. The 7-day moving average of the daily number of tests per 100,000 population varied greatly among countries, with Nigeria reporting 3 tests and Austria reporting 3,023 tests per 100,000 as of April 26, 2021 [26]. Countries with high test rates may report greater numbers of cases than countries with lower test rates, resulting in variation in reported incidence rates among countries. Additionally, the number of detected asymptomatic patients mainly depends on the level of testing; moreover, some studies have reported that the number of asymptomatic patients may vary based on age [27]. Countries that test asymptomatic patients in age groups with greater proportions of asymptomatic cases may also report higher incidence rates. In addition, COVID-19 is diagnosed using multiple methods (i.e., polymerase chain reaction and a rapid antigen test), which have different levels of sensitivity and specificity [28]. As a result, the incidence rates could vary according to the method of diagnosis. There are 2 main ways in which COVID-19 deaths are counted. Countries such as Belgium, Canada, and Germany define COVID-19 deaths as clinically confirmed or probable cases without laboratory confirmation, whereas countries such as Austria, Italy, and Spain count deaths due to laboratory-confirmed COVID-19 [29]. Consequently, mortality rates may be underestimated or overestimated based on the definition of COVID-19 deaths used. In addition, when post-mortem COVID-19 testing is not conducted, undocumented COVID-19 deaths can occur for various reasons, such as fear of sending a family member diagnosed with COVID-19 to die alone in an isolated facility [30] or limited testing capacity [31], resulting in the underestimation of COVID-19 deaths [32].

The positive correlation between incidence rates and the number of doctors per population and negative correlations between CFRs and GDP, the number of beds per population, and the number of doctors per population suggest that medical resources are important both for detecting COVID-19 cases and preventing COVID-19-related deaths. In Wuhan, China, the COVID-19 mortality rate was dependent on the timely supply of medical resources, including intensive care units and health workers [33]. In addition, an ecological analysis of 65 countries showed that preventing hospitals from being overrun contributed to flattening the COVID-19 epidemic curve [34].

The mortality rates were observed to be positively related to the number of doctors per population. In the early stage of the outbreak, the United States, European countries, and Republic of Korea recorded high mortality rates in long-term care facilities [35-37]. Subsequently, the fatality rate of the countries decreased with time. This is thought to be explainable on the basis of the following facts: (1) high mortality rates could exist only with high incidence rates; and (2) high incidence rates were associated with the number of doctors per population. In other words, higher mortality rates were subsequent results of higher incidence rates, which were associated with the number of doctors per population.

The R^2^ value between the incidence and mortality rates for individuals aged 60 years or older was higher than that for individuals of all ages. Moreover, in contrast to the analysis including all ages, the analysis of the relationship between the truncated CFR (age≥ 60) and the number of beds per population yielded a smaller absolute value of the Pearson correlation coefficient, suggesting a much weaker correlation. Even the analysis of the relationship between CFR and doctors per population among the elderly did not show any significant associations. This shows that there is little room for intervention to prevent COVID-19 deaths in the elderly and that preventing infections may be more effective than managing elderly COVID-19 patients. This indicates not only that COVID-19 vaccination is important for older people, but also that they should have a higher priority for the vaccine. Because COVID-19 is transmitted through direct contact with respiratory droplets and indirect contact with contaminated surfaces or spaces, preventing transmission in nursing homes or welfare facilities through periodic disinfection, ventilation, and adherence to quarantine guidelines may be important in preventing COVID-19 deaths among elderly individuals.

There are several limitations to our study. First, the interpretation of the data may be subject to the ecological fallacy, which is inherent in ecological studies; the true relationship at the individual level may differ from the results of our study. Second, data on COVID-19 were obtained from a snapshot on April 6, 2021, which does not reflect serial changes, and the abovementioned findings may have been captured by chance at that time. However, similar results of this study were observed using the data as of October 8, 2020 (Supplementary Material 3), suggesting that the global second wave of the pandemic did not affect the observed correlations. Third, although cases/deaths without age data were distributed to avoid the underestimation of age-standardized rates, this may have shifted the rates in the incorrect direction if cases/deaths with unknown age were not evenly distributed across the age range of interest. Finally, there may be other variables of interest, such as testing policies, the government response to COVID-19 (i.e., level of social distancing), and indicators of national health status, that were not considered in this study. For example, we could not reflect the effects of vaccination. After the United Kingdom started COVID-19 vaccination on December 8, 2020, it was followed by other countries [38]. As of April 6, 2021, 64 of the 79 countries included in this study have started to administer COVID-19 vaccines. The proportion of fully vaccinated people against COVID-19 was highest in Israel (56.44%) and lowest in Viet Nam (0.01%) at the time we collected the data [39]. However, we could not consider vaccination in our study for the following reasons: (1) there was a discrepancy in data collecting period between incidence and vaccination rates; and (2) only a few countries reported demographic data for vaccinated people. Therefore, further research on the effects of these factors is needed.

While reports on the clinical course of COVID-19 and the rapidly changing daily situation are abundant, discussions on public health management from a global perspective are limited [40]. We hope that our findings regarding the associations between age-standardized rates and GDP per capita, number of beds per population, and number of doctors per population across 79 countries can serve as a milestone in global public health.

In conclusion, age-standardized rates differed from crude rates because of discrepancies in the age structure of populations. Medical resources were associated with higher incidence rates and lower fatality rates. For those aged 60 years or more, not only were these associations weaker, but also the correlation between incidence and mortality rates was stronger than in the overall population. To minimize the burden caused by COVID-19, preventing infections in the elderly is much more important than treatment when considering the high fatality rate of COVID-19 in this group.

## Figures and Tables

**Table 1.** Cumulative crude and age-standardized incidence rates (per 100,000), mortality rates (per 100,000), and CFRs (%) of 79 countries as of April 6, 2021

Country		Age-standardized rates											
		All ages			Age≥60			All ages			Age≥60		
		Incidence	Mortality	CFR	Incidence	Mortality	CFR	Incidence	Mortality	CFR	Incidence	Mortality	CFR
Asia													
	Azerbaijan	2,562.53	NA	NA	4,758.19	NA	NA	2,681.03	36.60	1.37	4,837.18	NA	NA
	Hong Kong	145.58	1.15	0.85	151.85	8.47	6.41	153.52	2.73	1.78	149.35	9.78	6.55
	Indonesia	559.76	16.23	2.47	610.24	73.33	12.02	563.94	15.35	2.72	610.24	73.33	12.02
	Israel	9,966.25	45.49	0.71	6,181.43	325.21	5.38	9,646.08	72.29	0.75	6,178.22	402.09	6.51
	Japan	417.33	1.82	0.64	263.64	13.54	4.93	383.73	7.30	1.90	266.78	20.55	7.70
	Jordan	6,747.11	130.22	1.45	8,916.01	876.38	9.63	6,203.07	70.58	1.14	8,902.03	829.21	9.31
	Laos	0.69	0.00	0.00	0.44	0.00	0.00	0.67	0.00	0.00	0.40	0.00	0.00
	Lebanon	6,947.16	NA	NA	12,015.15	NA	NA	7,025.20	93.90	1.34	12,041.68	NA	NA
	Macao	8.76	0.00	0.00	2.12	0.00	0.00	7.39	0.00	0.00	2.45	0.00	0.00
	Malaysia	NA	4.41	NA	NA	26.49	NA	1,091.67	4.02	0.37	NA	25.26	NA
	Maldives	4,494.20	23.58	0.65	4,052.28	164.37	4.84	4,607.95	12.39	0.27	4,057.58	162.77	4.01
	Nepal	1,035.04	NA	NA	1,173.18	NA	NA	955.73	10.42	1.09	1,153.79	NA	NA
	Palestine	6,119.09	130.33	1.12	11,367.04	942.95	7.58	4,925.85	53.04	1.08	11,078.60	840.75	7.59
	Philippines	781.56	16.74	1.61	1,107.55	98.33	8.68	733.78	12.27	1.67	1,096.74	90.88	8.29
	Republic of Korea	189.26	1.65	0.68	245.22	12.84	5.24	207.20	3.42	1.65	245.79	14.04	5.71
	Sri Lanka	NA	2.20	NA	NA	12.56	NA	437.09	2.71	0.62	NA	12.05	NA
	Taiwan	6.18	0.09	1.48	7.27	0.50	9.39	5.99	0.06	0.95	7.49	0.46	6.08
	Thailand	43.91	0.09	0.63	11.15	0.37	4.12	42.37	0.14	0.32	11.13	0.37	3.29
	Viet NAm	2.61	0.03	1.42	2.09	0.17	8.76	2.72	0.04	1.32	2.10	0.02	8.73
Europe													
	Albania	3,970.89	49.81	0.89	5,406.47	280.12	5.17	4,405.97	79.02	1.79	5,407.68	283.49	5.24
	Austria	6,167.91	36.48	0.64	5,234.91	280.85	4.97	6,250.08	105.67	1.69	5,371.85	394.53	7.34
	Belgium	7,624.14	68.17	0.82	6,306.69	503.89	6.10	7,791.14	200.20	2.57	6,971.95	743.94	10.67
	Bosnia and HerzegoviNA	4,780.14	21.86	0.39	5,574.23	148.56	2.67	5,377.10	211.93	3.94	5,568.65	150.41	2.70
	Bulgaria	4,989.86	NA	NA	5,882.31	NA	NA	5,080.26	198.40	3.86	6,032.67	NA	NA
	Croatia	6,605.39	NA	NA	5,630.64	NA	NA	6,842.41	148.91	2.18	5,597.38	NA	NA
	Cyprus	3,728.47	NA	NA	3,217.69	NA	NA	3,798.70	21.20	0.56	3,183.02	NA	NA
	Czechia	14,252.80	105.75	0.82	12,777.85	779.31	6.27	14,522.81	253.70	1.75	12,519.55	908.72	7.26
	Denmark	4,315.03	15.13	0.59	2,507.65	118.71	4.76	4,036.41	41.95	1.04	2,451.18	156.05	6.37
	Estonia	8,236.66	NA	NA	7,163.97	NA	NA	8,343.54	72.67	0.87	7,020.41	NA	NA
	Faroe Islands	1,490.02	NA	NA	653.57	NA	NA	1,352.76	2.05	0.15	653.89	NA	NA
	Finland	1,647.65	5.23	0.88	592.64	38.18	6.98	1,439.11	15.27	1.06	573.98	50.07	8.72
	France	NA	49.68	NA	NA	367.50	NA	7,388.10	148.11	2.00	NA	526.36	NA
	Germany	3,437.10	27.06	0.83	2,732.16	207.80	6.44	3,469.05	92.76	2.67	2,995.15	313.21	10.46
	Greece	2,583.88	34.77	1.57	2,330.00	222.74	10.94	2,660.23	81.10	3.05	2,240.82	246.46	11.00
	Hungary	NA	107.81	NA	NA	738.29	NA	7,204.88	230.16	3.19	NA	784.95	NA
	Ireland	4,893.14	NA	NA	4,353.63	NA	NA	4,838.34	95.73	1.98	4,438.43	NA	NA
	Isle of Man	2,215.13	NA	NA	635.14	NA	NA	1,847.52	34.10	1.85	580.84	NA	NA
	Italy	5,909.96	54.25	0.91	5,508.50	405.46	6.90	6,084.74	184.13	3.03	5,721.36	589.89	10.31
	Latvia	5,110.11	42.07	0.77	5,443.31	277.74	5.36	5,542.00	102.53	1.85	5,399.37	331.07	6.13
	Lithuania	NA	52.04	NA	NA	358.64	NA	8,114.50	133.09	1.64	NA	438.82	NA
	Luxembourg	10,145.48	57.52	0.58	7,818.05	451.99	4.59	10,004.35	121.58	1.22	8,484.74	586.74	6.92
	Moldova	4,903.12	89.68	1.04	9,688.52	581.08	6.51	5,845.12	128.36	2.20	9,748.60	546.47	5.61
	Montenegro	13,865.56	NA	NA	14,973.34	NA	NA	14,812.14	211.12	1.43	14,928.85	NA	NA
	Netherlands	8,063.76	34.64	0.50	5,703.58	272.42	3.94	7,662.78	97.16	1.27	6,284.48	355.32	5.65
	North Macedonia	5,691.33	NA	NA	7,523.18	NA	NA	6,487.89	190.89	2.94	7,523.18	NA	NA
	Norway	1,956.45	5.28	0.61	879.06	38.93	4.82	1,820.14	12.65	0.70	856.79	51.22	5.98
	Poland	NA	70.50	NA	NA	493.90	NA	6,491.23	145.50	2.24	NA	533.93	NA
	Portugal	8,147.96	51.15	0.67	6,597.39	385.58	5.15	8,084.65	165.61	2.05	6,915.54	541.57	7.83
	Romania	4,441.14	NA	NA	5,607.70	NA	NA	5,110.89	126.76	2.48	5,568.14	NA	NA
	Slovakia	6,328.62	97.76	1.51	5,935.49	693.11	11.14	6,690.05	184.89	2.76	5,956.87	713.05	11.97
	Slovenia	9,915.74	65.88	0.63	9,175.13	507.16	4.93	10,615.90	196.69	1.85	9,434.86	683.23	7.24
	Spain	7,420.28	55.30	0.90	5,708.87	413.46	6.84	7,082.32	162.09	2.29	5,822.35	586.70	10.08
	Sweden	8,422.31	44.63	0.83	5,489.14	340.10	6.59	8,267.86	134.00	1.62	5,317.57	497.86	9.36
	Switzerland	6,745.08	41.71	0.57	6,080.47	332.58	4.57	7,051.42	120.14	1.70	6,333.68	464.57	7.33
	United Kingdom	6,440.57	73.87	1.39	4,716.52	527.21	10.44	6,429.20	186.90	2.91	4,898.19	712.62	14.55
Oceania													
	Australia	112.27	1.38	1.00	103.02	11.01	7.85	115.15	3.56	3.10	111.22	16.01	14.39
	Fiji	9.38	0.23	2.23	7.38	1.96	18.63	8.08	0.24	2.99	8.11	2.32	28.57
	New Zealand	52.99	0.26	0.90	35.74	1.75	6.80	52.34	0.54	1.03	34.26	2.15	6.27
North America													
	Bermuda	2,362.55	8.58	0.47	1,573.95	52.15	3.01	2,247.99	19.27	0.86	1,618.85	58.27	3.60
	Canada	2,712.34	24.43	0.90	2,048.61	187.09	6.90	2,687.64	61.25	2.28	2,155.12	231.11	10.72
	El Salvador	1,039.69	NA	NA	1,287.97	NA	NA	1,009.70	31.30	3.10	1,282.73	NA	NA
	Guatemala	1,272.19	59.45	3.42	1,812.92	307.23	17.36	1,092.23	38.48	3.52	1,807.57	308.82	17.08
	Haiti	129.00	3.11	1.99	216.80	15.30	6.86	112.28	2.20	1.96	215.86	14.72	6.82
	Jamaica	1,344.19	18.60	0.98	1,892.92	107.39	5.38	1,385.03	20.87	1.51	1,908.64	112.34	5.89
	Mexico	1,752.95	182.10	6.66	2,910.89	992.41	34.05	1,746.42	174.47	9.99	2,901.01	985.63	33.98
	Panama	8,279.68	134.20	1.38	9,543.01	831.26	8.66	8,263.62	142.26	1.72	9,556.16	875.28	9.16
	USA	9,309.72	86.86	0.97	8,647.11	546.33	6.39	9,516.65	172.01	1.81	8,670.58	643.98	7.43
South America													
	Argentina	5,272.62	100.91	1.83	5,230.34	676.78	12.94	5,326.07	124.95	2.35	5,230.74	676.78	12.94
	Chile	5,808.45	84.48	1.49	5,527.17	564.02	10.37	6,118.72	123.69	2.16	5,530.17	600.85	10.86
	Colombia	4,630.26	114.08	1.90	5,812.59	740.52	12.24	4,827.57	125.96	2.61	5,829.74	750.95	12.88
	Ecuador	1,957.37	103.92	3.50	3,184.82	610.15	19.05	1,908.84	96.28	5.04	3,184.93	610.84	19.18
	Peru	4,662.47	152.92	2.33	6,800.50	879.19	12.96	4,799.15	160.37	3.34	6,801.61	890.25	13.09
	Venezuela	596.32	NA	NA	427.63	NA	NA	584.20	5.84	1.00	432.26	NA	NA
Africa													
	Cabo Verde	3,288.13	44.82	0.84	4,809.11	330.02	5.74	3,226.51	31.12	0.96	4,776.30	345.07	7.22
	Eswatini	1,984.58	112.45	3.71	3,128.65	611.38	19.83	1,494.18	57.66	3.86	3,085.37	606.06	19.64
	Gambia	351.87	NA	NA	734.50	NA	NA	228.19	6.88	3.02	730.79	NA	NA
	Mozambique	306.37	5.83	1.45	376.54	32.31	8.58	218.29	2.50	1.15	376.54	32.31	8.58
	Nigeria	113.26	2.64	1.44	177.02	16.20	8.35	79.17	1.00	1.26	159.11	12.58	7.91
	Togo	181.71	2.87	1.13	298.97	15.42	5.07	135.13	1.35	1.00	291.16	13.41	4.61

CFR, case fatality rate; NA, not available.

**Table 2.** Pearson correlation coefficients among the log-transformed variables

Variables	All ages						Age≥60					
	Incidence	p-value	Mortality	p-value	CFR	p-value	Incidence	p-value	Mortality	p-value	CFR	p-value
GDP per capita	0.27	0.019	0.09	0.498	-0.54	<0.001	0.14	0.254	0.13	0.301	-0.36	0.006
No. of beds	0.24	0.037	0.00	0.979	-0.56	<0.001	0.19	0.115	0.04	0.737	-0.41	0.002
No. of doctor	0.47	<0.001	0.29	0.023	-0.37	0.005	0.37	0.001	0.33	0.009	-0.18	0.194

All variables were log-transformed. CFR, case fatality rate; GDP, gross domestic product.

## SUPPLEMENTARY MATERIALS

Supplementary Material 1. Sources of data for cases and deaths

Supplementary Material 2. Methods: Exceptions to rules with regard to cases or deaths of COVID-19.

Supplementary Material 3. Pearson's correlation coefficients and partial correlation coefficients among the log-transformed variables as of October 8, 2020. (n=55)
