# Supplementary material for: The age-standardized incidence, mortality, and case fatality rates of COVID-19 in 79 countries: a cross-sectional comparison and their correlations with associated factors
Source: Epidemiol Health. 2021 Sep 8;43:e2021061. doi: 10.4178/epih.e2021061 (PMC8611321; doi:10.4178/epih.e2021061)
Supplement: Supplementary Material 1. — Sources of data for cases and deaths [file epih-43-e2021061-suppl1.docx]

Supplementary Material 1. Sources of data for cases and deaths

|  | Source of cases | Source of deaths |
| --- | --- | --- |
| Albania | Agjencia Kombëtare e Shoqerisë së Informacionit [1] | Agjencia Kombëtare e Shoqerisë së Informacionit [1] |
| Argentina | Ministerio de Salud [2] | Ministerio de Salud [2] |
| Australia | Department of Health [3] | Department of Health [3] |
| Austria | Agentur für Gesundheit und Ernährungssicherheit GmbH (AGES) [4] | Cooperation OGD Österreich [5] |
| Azerbaijan | Nazirlər Kabineti yanında Operativ Qərargah [6] | Not Provided |
| Belgium | Sciensano [7] | Sciensano [7] |
| Bermuda | Government of Bermuda [8] | Government of Bermuda [9] |
| Bosnia and Herzegovina | Zavod Za Javno Zdravstvo FBiH [10] | Zavod Za Javno Zdravstvo FBiH [10] |
| Bulgaria | Министерство на здравеопазването [11] | Not Provided |
| Cabo Verde | Governo de Cabo Verde [12] | Governo de Cabo Verde [12] |
| Canada | Government of Canada [13] | Government of Canada [13] |
| Chile | Ministerio de Salud [14] | Ministerio de Salud [14] |
| Colombia | Ministerio de Salud y Protección Social [15] | Ministerio de Salud y Protección Social [15] |
| Croatia | Hrvatski Zavod Za Javno Zdravstvo [16] | Not Provided |
| Cyprus | Υπουργείο Υγείας, Κυπριακή Δημοκρατία [17] | Υπουργείο Υγείας, Κυπριακή Δημοκρατία [17] |
| Czechia | Ministerstvo zdravotnictví České republiky [18] | Ministerstvo zdravotnictví České republiky [18] |
| Denmark | Sundhedsstyrelsen [19] | Statens Serum Institut [20] |
| Ecuador | Ministerio de Salud Pública [21] | Ministerio de Salud Pública [21] |
| El Salvador | Gobierno de El Salvador [22] | Not Provided |
| Estonia | Terviseamet [23] | Not Provided |
| Eswatini | The Government of the Kingdom of Eswatini [24] | The Government of the Kingdom of Eswatini [24] |
| Faroe Islands | Føroya Landsstýri [25] | Not Needed (deaths = 0) |
| Fiji | Ministry of Health & Medical Services [26] | News articles [27] |
| Finland | Terveyden ja hyvinvoinnin laitos / National Infectious Disease Register [28] | Terveyden ja hyvinvoinnin laitos [28] |
| France | Not Provided | Esri France [29] |
| Gambia | Ministry of Health, the Gambia [30] | Not Provided |
| Germany | Robert Koch Institut [31] | Robert Koch Institut [32] |
| Greece | ΕΛΛΗΝΙΚΗ ΔΗΜΟΚΡΑΤΙΑ Κυβέρνηση [33] | ΕΛΛΗΝΙΚΗ ΔΗΜΟΚΡΑΤΙΑ Κυβέρνηση [33] |
| Guatemala | Ministerio de Salud Pública y Asistencia Social [34] | Ministerio de Salud Pública y Asistencia Social [34] |
| Haiti | Ministère de la Santé Publique et de la Population [35] | Ministère de la Santé Publique et de la Population [35] |
| Hong Kong | Centre for Health Protection of Department of Health [36] | Centre for Health Protection of Department of Health [36] |
| Hungary | Not Provided | Nemzeti Népegészségügyi Központ [37] |
| Indonesia | Komite Penanganan COVID-19 Dan Pemjulihan Ekonomi Nasional [38] | Komite Penanganan COVID-19 Dan Pemjulihan Ekonomi Nasional [38] |
| Ireland | Health Protection Surveillance Centre [39] | Not Provided |
| Isle of Man | Isle of Man Government [40] | Not Provided |
| Israel | Israel Mnistry of Health [41] | Not Provided |
| Italy | EpiCentro - Istituto Superiore di Sanità [42] | EpiCentro - Istituto Superiore di Sanità [42] |
| Jamaica | Ministry of Health & Wellness Jamaica [43] | Ministry of Health & Wellness Jamaica [43] |
| Japan | Ministry of Health, Labor and Welfare [44] | Ministry of Health, Labor and Welfare [44] |
| Jordan | Ministry of Health [45] | Not Provided |
| Laos | Ministry of health, news articles [46] | Not Needed (deaths = 0) |
| Latvia | Slimību profilakses un kontroles centrs [47] | Slimību profilakses un kontroles centrs [47] |
| Lebanon | Lebanese Ministry of Information [48] | Not Provided |
| Lithuania | Not Provided | Oficialiosios statistikos portalas [49] |
| Luxembourg | Le gouvernement luxembourgeois [50] | Le gouvernement luxembourgeois [50] |
| Macao | Centre for Disease Control and Prevention (CDC) [51] | Not Needed (deaths = 0) |
| Malaysia | Not Provided | ESRI Malaysia [52] |
| Maldives | Ministry of Health [53] | News articles [54] |
| Mexico | La Secretaría de Salud [55] | La Secretaría de Salud [55] |
| Moldova | Ministerului Sănătății, Muncii și Protecției Sociale [56] | Ministerului Sănătății, Muncii și Protecției Sociale [56] |
| Montenegro | Instituta za javno zdravlje Crne Gore [57] | Not Provided |
| Mozambique | Ministério da Saúde (MISAU) [58] | Ministério da Saúde (MISAU) [58] |
| Nepal | Ministry of Health and Population [59] | Not Provided |
| Netherlands | Rijksinstituut voor Volksgezondheid en Milieu [60] | Rijksinstituut voor Volksgezondheid en Milieu [61] |
| New Zealand | Ministry of Health [62] | Ministry of Health [62] |
| Nigeria | Nigeria Centre for Disease Control [63] | Nigeria Centre for Disease Control [63] |
| North Macedonia | Влада на Република Северна Македонија [64] | Not Provided |
| Norway | Norwegian Institute of Public Health [65] | Norwegian Institute of Public Health [65] |
| Palestine | Palestinian National Institute of Public Health [66] | Palestinian National Institute of Public Health [66] |
| Panamá | Ministerio de Salud [67] | Ministerio de Salud [67] |
| Peru | Instituto Nacional de Salud y Centro Nacional de Epidemiologia, prevención y Control de Enfermedades – MINSA [68] | Instituto Nacional de Salud y Centro Nacional de Epidemiologia, prevención y Control de Enfermedades – MINSA [69] |
| Philippines | Department of Health [70] | Department of Health [70] |
| Poland | Ministerstwo Zdrowia [71] | Ministerstwo Zdrowia [71] |
| Portugal | Direção-Geral da Saúde [72] | Direção-Geral da Saúde [73] |
| Republic of Korea | Ministry of Health and Welfare [74] | Ministry of Health and Welfare [74] |
| Romania | Guvernul României [75] | Not Provided |
| Slovakia | Institut-Zdravotnych-Analyz [76] | Institut-Zdravotnych-Analyz [77] |
| Slovenia | Nacionalni inštitut za javno zdravje [78] | Nacionalni inštitut za javno zdravje [78] |
| Spain | Instituto de Salud Carlos III [79] | Instituto de Salud Carlos III [79] |
| Sri Lanka | Not Provided | Ministry of Health [80] |
| Sweden | Folkhälsomyndigheten [81] | Folkhälsomyndigheten [81] |
| Switzerland | Federal Office of Public Health [82] | Federal Office of Public Health [82] |
| Taiwan | Ministry of Health and Welfare [83] | News articles |
| Thailand | Department of Disease Control [84] | News articles |
| Togo | République Togolaise [85] | République Togolaise [85] |
| United Kingdom (UK) - England | Public Health England [86] | Public Health England [87] |
| United Kingdom (UK) - Wales | Public Health Wales [88] | Public Health Wales [89] |
| United Kingdom (UK) - Northern Ireland | Department of Health [90] | Department of Health [90] |
| United Kingdom (UK) - Scotland | Public Health Scotland [91] | Public Health Scotland [91] |
| United States of America (USA) | Centers for Disease Control and Prevention [92] | Centers for Disease Control and Prevention [92] |
| Venezuela | La Plataforma Patria [93] | Not Provided |
| Viet Nam | Bộ Y tế [94] | Bộ Y tế [94] |

References

1. https://coronavirus.al/statistika/, Accessed Apr 06, 2021.

2. https://www.argentina.gob.ar/salud/coronavirus-COVID-19/sala-situacion, Accessed Apr 06, 2021.

3.https://www.health.gov.au/news/health-alerts/novel-coronavirus-2019-ncov-health-alert/coronavirus-covid-19-current-situation-and-case-numbers#cases-and-deaths-by-age-and-sex, Accessed Apr 06, 2021.

4. https://covid19-dashboard.ages.at/?l=en, Accessed Apr 06, 2021.

5.https://www.data.gv.at/katalog/dataset/covid-19-daten-covid19-faelle-je-altergruppe/resource/9b11f7a9-9cec-49bb-89ad-6452f4f72172, Accessed Apr 06, 2021.

6. https://koronavirusinfo.az/az/page/statistika/azerbaycanda-cari-veziyyet, Accessed Apr 06, 2021.

7.https://datastudio.google.com/embed/u/0/reporting/c14a5cfc-cab7-4812-848c-0369173148ab/page/tpRKB, Accessed Apr 06, 2021.

8.https://app.powerbi.com/view?r=eyJrIjoiYTVjY2MyY2YtZmEzNy00NDhjLWJkMjEtNGFjMjM0MmFmOWFmIiwidCI6IjAzNTQ4YjRhLTRmNjQtNDc0Ny1iNDdjLTcwODY5ZDE2NWMzZCIsImMiOjF9,Accessed Apr 06, 2021.

9. https://www.gov.bm/coronavirus-covid19-update, Accessed Apr 06, 2021.

10. https://covid-19.ba/, Accessed Apr 06, 2021.

11. https://coronavirus.bg/bg/statistika, Accessed Apr 06, 2021.

12. https://covid19.cv/, Accessed Apr 06, 2021.

13. https://health-infobase.canada.ca/covid-19/epidemiological-summary-covid-19-cases.html, Accessed Apr 06, 2021.

14. https://www.gob.cl/coronavirus/cifrasoficiales/, Accessed Apr 06, 2021.

15.https://app.powerbi.com/view?r=eyJrIjoiMjBjZWNlOGUtNzc1Yi00NjVkLTkyMjktOTJmMGU3YTU2Nzk4IiwidCI6ImE2MmQ2YzdiLTlmNTktNDQ2OS05MzU5LTM1MzcxNDc1OTRiYiIsImMiOjR9&pageName=ReportSection0c50ea3406afe4407370, Accessed Apr 06, 2021.

16. https://www.koronavirus.hr/podaci/489, Accessed Apr 06, 2021.

17. https://covid19.ucy.ac.cy/, Accessed Apr 06, 2021.

18. https://onemocneni-aktualne.mzcr.cz/covid-19, Accessed Apr 06, 2021.

19. https://www.sst.dk/en/English/Corona-eng/Status-of-the-epidemic/COVID-19-updates-Statistics-and-charts, Accessed Apr 06, 2021.

20. https://experience.arcgis.com/experience/aa41b29149f24e20a4007a0c4e13db1d, Accessed Apr 06, 2021.

21.https://public.tableau.com/profile/direcci.n.nacional.de.vigilancia.epidemiol.gica.msp#!/vizhome/COVID19ecu_MSP_DNVE/COVID-19MSP, Accessed Apr 06, 2021.

22. https://covid19.gob.sv/, Accessed Apr 06, 2021.

23. https://koroonakaart.ee/et, Accessed Apr 06, 2021.

24. https://datastudio.google.com/embed/u/0/reporting/b847a713-0793-40ce-8196-e37d1cc9d720/page/2a0LB, Accessed Apr 06, 2021.

25. https://corona.fo/statistics?_l=en, Accessed Apr 06, 2021.

26. http://www.health.gov.fj/covid-19-updates/, Accessed Apr 06, 2021.

27. <http://www.xinhuanet.com/english/2020-08/25/c_139316785.htm>, Accessed Apr 06, 2021.

28. https://experience.arcgis.com/experience/92e9bb33fac744c9a084381fc35aa3c7, Accessed Apr 06, 2021.

29.https://www.arcgis.com/apps/opsdashboard/index.html#/5e09dff7cb434fb194e22261689e2887, Accessed Apr 06, 2021.

30. http://www.moh.gov.gm/covid-19-report/, Accessed Apr 06, 2021.

31.https://www.rki.de/DE/Content/InfAZ/N/Neuartiges_Coronavirus/Daten/Altersverteilung.html;jsessionid=FDFFF38C0ABEE63D78FAF070681A9963.internet122?nn=13490888, Accessed Apr 06, 2021.

32.https://www.rki.de/DE/Content/InfAZ/N/Neuartiges_Coronavirus/Projekte_RKI/COVID-19_Todesfaelle.html;jsessionid=11D8A3BAB128687F6C30C58A4E51F548.internet121?nn=13490888, Accessed Apr 06, 2021.

33. https://covid19.gov.gr/covid19-live-analytics/, Accessed Apr 06, 2021.

34. https://tablerocovid.mspas.gob.gt/, Accessed Apr 06, 2021.

35. https://www.mspp.gouv.ht/documentation/, Accessed Apr 06, 2021.

36. https://www.chp.gov.hk/en/features/102997.html, Accessed Apr 06, 2021.

37. https://koronavirus.gov.hu/elhunytak, Accessed Apr 06, 2021.

38. https://covid19.go.id/peta-sebaran, Accessed Apr 06, 2021.

39. https://covid19ireland-geohive.hub.arcgis.com/pages/detailed-profile-of-cases, Accessed Apr 06, 2021.

40. https://covid19.gov.im/general-information/latest-updates/, Accessed Apr 06, 2021.

41.https://datadashboard.health.gov.il/COVID-19/general?utm_source=go.gov.il&utm_medium=referral, Accessed Apr 06, 2021.

42. https://www.epicentro.iss.it/coronavirus/sars-cov-2-dashboard/, Accessed Apr 06, 2021.

43. https://jamcovid19.moh.gov.jm/, Accessed Apr 06, 2021.

44. https://toyokeizai.net/sp/visual/tko/covid19/en.html, Accessed Apr 06, 2021.

45. https://corona.moh.gov.jo/en, Accessed Apr 06, 2021.

46. http://www.xinhuanet.com/english/2021-03/13/c_139807839.htm, Accessed Apr 06, 2021

47. https://www.spkc.gov.lv/lv/aktualitates-par-covid-19, Accessed Apr 06, 2021

48. https://corona.ministryinfo.gov.lb/, Accessed Apr 06, 2021

49. https://osp.stat.gov.lt/praejusios-paros-covid-19-statistika, Accessed Apr 06, 2021

50. https://covid19.public.lu/fr/graph.html#tc, Accessed Apr 06, 2021

51. https://www.ssm.gov.mo/apps1/PreventCOVID-19/en.aspx#clg17458, Accessed Apr 06, 2021

52. https://experience.arcgis.com/experience/e4e031b3046f479d8812331d6e6dd665, Accessed Apr 06, 2021

53. https://covid19.health.gov.mv/dashboard/, Accessed Apr 06, 2021

54. https://edition.mv/covid_19/, Accessed Apr 06, 2021

55. https://datos.covid-19.conacyt.mx/#DOView, Accessed Apr 06, 2021

56.https://gismoldova.maps.arcgis.com/apps/opsdashboard/index.html#/d274da857ed345efa66e1fbc959b021b, Accessed Apr 06, 2021

57. https://www.covidodgovor.me/me/statistika, Accessed Apr 06, 2021

58. https://covid19.ins.gov.mz/documentos-em-pdf/sumario-epidemiologico/, Accessed Apr 06, 2021

59. https://covid19.mohp.gov.np/, Accessed Apr 06, 2021

60. https://coronadashboard.rijksoverheid.nl/landelijk/positief-geteste-mensen#ggd, Accessed Apr 06, 2021

61. https://www.rivm.nl/coronavirus-covid-19/grafieken, Accessed Apr 06, 2021

62.https://www.health.govt.nz/our-work/diseases-and-conditions/covid-19-novel-coronavirus/covid-19-data-and-statistics/covid-19-case-demographics, Accessed Apr 06, 2021

63.https://ncdc.gov.ng/diseases/sitreps/?cat=14&name=An%20update%20of%20COVID-19%20outbreak%20in%20Nigeria, Accessed Apr 06, 2021

64. https://koronavirus.gov.mk/stat, Accessed Apr 06, 2021

65.https://www.fhi.no/en/id/infectious-diseases/coronavirus/daily-reports/daily-reports-COVID19/, Accessed Apr 06, 2021

66. http://site.moh.ps/index/covid19/LanguageVersion/0/Language/ar, Accessed Apr 06, 2021

67. http://www.minsa.gob.pa/informacion-salud/presentaciones-covid-19-detalles, Accessed Apr 06, 2021

68. https://www.datosabiertos.gob.pe/dataset/casos-positivos-por-covid-19-ministerio-de-salud-minsa, Accessed Apr 06, 2021

69. https://covid19.minsa.gob.pe/sala_situacional.asp, Accessed Apr 06, 2021

70. https://ncovtracker.doh.gov.ph/, Accessed Apr 06, 2021

71. https://experience.arcgis.com/experience/d332e064cd4a476198a007c256dcf5bc, Accessed Apr 06, 2021

72. https://covid19.min-saude.pt/ponto-de-situacao-atual-em-portugal/, Accessed Apr 06, 2021

73. https://covid19.min-saude.pt/relatorio-de-situacao/, Accessed Apr 06, 2021

74.http://ncov.mohw.go.kr/bdBoardList_Real.do?brdId=1&brdGubun=11&ncvContSeq=&contSeq=&board_id=&gubun=, Accessed Apr 06, 2021

75. https://datelazi.ro/, Accessed Apr 06, 2021

76. https://github.com/Institut-Zdravotnych-Analyz/covid19-data/tree/main/PCR_Tests, Accessed Apr 06, 2021

77. https://github.com/Institut-Zdravotnych-Analyz/covid19-data/tree/main/Deaths, Accessed Apr 06, 2021

78. https://covid-19.sledilnik.org/sl/stats, Accessed Apr 06, 2021

79.https://www.isciii.es/QueHacemos/Servicios/VigilanciaSaludPublicaRENAVE/EnfermedadesTransmisibles/Paginas/InformesCOVID-19.aspx, Accessed Apr 06, 2021

80.http://www.epid.gov.lk/web/index.php?option=com_content&view=article&id=225&Itemid=518&lang=en, Accessed Apr 06, 2021

81. https://experience.arcgis.com/experience/09f821667ce64bf7be6f9f87457ed9aa/page/page_0/, Accessed Apr 06, 2021

82. https://www.bag.admin.ch/bag/de/home/krankheiten/ausbrueche-epidemien-pandemien/aktuelle-ausbrueche-epidemien/novel-cov/situation-schweiz-und-international.html, Accessed Apr 06, 2021

83. https://covid19.mohw.gov.tw/ch/cp-4707-52357-205.html, Accessed Apr 06, 2021

84. https://data.go.th/dataset/covid-19-daily, Accessed Apr 06, 2021

85. https://covid19.gouv.tg/graph-evolution/, Accessed Apr 06, 2021

86. https://coronavirus.data.gov.uk/details/cases?areaType=nation&areaName=England, Accessed Apr 06, 2021

87. https://coronavirus.data.gov.uk/details/deaths?areaType=nation%26areaName=England, Accessed Apr 06, 2021

88.https://public.tableau.com/profile/public.health.wales.health.protection#!/vizhome/RapidCOVID-19virology-Public/Summary, Accessed Apr 06, 2021

89.https://public.tableau.com/profile/public.health.wales.health.protection#!/vizhome/RapidCOVID-19virology-Public/PHWdeaths, Accessed Apr 06, 2021

90.https://app.powerbi.com/view?r=eyJrIjoiZGYxNjYzNmUtOTlmZS00ODAxLWE1YTEtMjA0NjZhMzlmN2JmIiwidCI6IjljOWEzMGRlLWQ4ZDctNGFhNC05NjAwLTRiZTc2MjVmZjZjNSIsImMiOjh9, Accessed Apr 06, 2021

91.https://www.opendata.nhs.scot/dataset/covid-19-in-scotland/resource/19646dce-d830-4ee0-a0a9-fcec79b5ac71?inner_span=True, Accessed Apr 06, 2021

92. https://covid.cdc.gov/covid-data-tracker/#demographics, Accessed Apr 06, 2021

93. https://covid19.patria.org.ve/estadisticas-venezuela/, Accessed Apr 06, 2021

94. https://ncov.moh.gov.vn/, Accessed Apr 06, 2021
