# Supplementary material for: The age-standardized incidence, mortality, and case fatality rates of COVID-19 in 79 countries: a cross-sectional comparison and their correlations with associated factors
Source: Epidemiol Health. 2021 Sep 8;43:e2021061. doi: 10.4178/epih.e2021061 (PMC8611321; doi:10.4178/epih.e2021061)
Supplement: Supplementary Material 2. — Methods: Exceptions to rules with regard to cases or deaths of COVID-19. [file epih-43-e2021061-suppl2.docx]

Supplementary Material 2. Methods: Exceptions to rules with regard to cases or deaths of COVID-19.

1. Belgium: Because age groups varied between cases and deaths, uniform age distributions among cases and deaths was required for calculation of CFR. Transforming age groups for cases to match the age groups for deaths was needed, however age groups was totally different between cases and deaths (cases: 0-9, 10-19, 20-29, 30-39, 40-49, 50-59, 60-69, 70-79, 80-89, 90-, deaths: 0-24, 25-44, 45-64, 65-74, 75-84, 85-). Therefore, cases were redistributed according to age group of deaths and the method is as follows:

| Age group | Number of cases |
| --- | --- |
| 0-24 | $C(0,9)+C(10,19)+\frac{P(20,24)}{P(20,29)}\times C(20,29)$ |
| 25-44 | $\frac{P\left( 25,29 \right)}{P\left( 20,29 \right)}\times C\left( 20,29 \right)+C(30,39)+\frac{P\left( 40,44 \right)}{P\left( 40,49 \right)}\times C(40,49)$ |
| 45-59 | $\frac{P\left( 45,49 \right)}{P\left( 40,49 \right)}\times C\left( 40,49 \right)+C(50,59)$ |
| 60-64 | $\frac{P\left( 60,64 \right)}{P\left( 60,69 \right)}\times C\left( 60,69 \right)$ |
| 65-74 | $\frac{P\left( 65,69 \right)}{P\left( 60,69 \right)}\times C\left( 60,69 \right)+\frac{P\left( 70,74 \right)}{P\left( 70,79 \right)}\times C(70,79)$ |
| 75-84 | $\frac{P\left( 75,79 \right)}{P\left( 70,79 \right)}\times C(70,79)+\frac{P\left( 80,84 \right)}{P\left( 80,89 \right)}\times C\left( 80,89 \right)$ |
| 85- | $\frac{P\left( 85,89 \right)}{P\left( 80,89 \right)}\times C\left( 80,89 \right)+C(90,\infty)$ |

where $C\left( a,b \right)$is the number of cases among age group $a-b$, and $P\left( c,d \right)$is the number of populations among age group $c-d$.

1. Finland: No exact data was provided about the death among age group 20 – 29, 30 – 39, and 40 – 49; to protect the privacy of deceased person, the number of deaths among them was represented as "<5" rather than exact number. It meant that there were 1 – 4 deaths of each group. As the difference between the total number of deaths and the sum of all deaths for each age group was 6 deaths, 2 deaths were evenly allocated for each age group. It only could be done under the assumption, there were no deaths of unknown age. It was plausible because there was no case with unknown age where the data of cases was more complete than that of deaths.
2. Gambia: The age groups of cases were 0-20, 21-30, 31-40, 41-50, 51-60, 61-70, 71-. To calculate the truncated rates (age ≥ 60), the number of cases aged 60 was needed. Therefore, the number of cases aged 60 was arbitrarily estimated as one tenth of the number of cases ages 51-60.
3. UK –The age group for cases and deaths was unified as 0-9, 10-19, 20-29, 30-39, 40-49, 50-59, 60-69, 70-79, 80. There was no problem for England and Wales, but Northern Ireland, and Scotland needed conversion.
   1. In the case of Northern Ireland, the age group had 20-year range; 0-19, 20-39, 40-59, 60-79, 80-. Therefore, they were divided proportionally according to the population distribution and specific method is as follows:

| Age group | Number of cases |
| --- | --- |
| 0-9 | $\frac{P(0,9)}{P(0,19)}\times C(0,19)$ |
| 10-19 | $\frac{P(10,19)}{P(0,19)}\times C(0,19)$ |
| 20-29 | $\frac{P(20,29)}{P(20,39)}\times C(20,39)$ |
| 30-39 | $\frac{P(30,39)}{P(20,39)}\times C(20,39)$ |
| 40-49 | $\frac{P(40,49)}{P(40,59)}\times C(40,59)$ |
| 50-59 | $\frac{P(50,59)}{P(40,59)}\times C(40,59)$ |
| 60-69 | $\frac{P(60,69)}{P(60,79)}\times C(60,79)$ |
| 70-79 | $\frac{P(70,79)}{P(60,79)}\times C(60,79)$ |
| 80- | $C(80,\infty)$ |

where $C\left( a,b \right)$is the number of cases among age group $a-b$, and $P\left( c,d \right)$is the number of populations among age group $c-d$.

- 1. Scotland had more complicated range and they were also divided proportionally according to the population distribution and specific method is as follows:

| Age group | Number of cases |
| --- | --- |
| 0-9 | $C\left( 0,4 \right)+\frac{P(5,9)}{P(5,14)}\times C(5,14)$ |
| 10-19 | $\frac{P(10,14)}{P(5,14)}\times C\left( 5,14 \right)+C\left( 15,19 \right)$ |
| 20-29 | $C\left( 20,24 \right)+\frac{P(25,29)}{P(25,44)}\times C(25,44)$ |
| 30-39 | $\frac{P(30,39)}{P(25,44)}\times C(25,44)$ |
| 40-49 | $\frac{P(40,44)}{P(25,44)}\times C\left( 25,44 \right)+\frac{P(45,49)}{P(45,64)}\times C\left( 45,64 \right)$ |
| 50-59 | $\frac{P(50,59)}{P(45,64)}\times C\left( 45,64 \right)$ |
| 60-69 | $\frac{P(60,64)}{P(45,64)}\times C\left( 45,64 \right)+\frac{P(65,69)}{P(65,74)}\times C\left( 65,74 \right)$ |
| 70-79 | $\frac{P(70,74)}{P(65,74)}\times C\left( 65,74 \right)+\frac{P(75,79)}{P(75,84)}\times C\left( 75,84 \right)$ |
| 80- | $\frac{P(80,84)}{P(75,84)}\times C\left( 75,84 \right)+C(85,\infty)$ |

Exceptions with regard to population or standard population

1. As World Health Organization (WHO) world standard population distribution 2000-2025 and United Nations Population Division’s ‘World Population Prospects: The 2019 Revision.' provided the population data as 5-year range (i.e. 0-4, 5-9, 10-14, 15-19, ...), specific conversions were done for the countries that showed the discrepancy with regard to age groups.
   1. Gambia: As the age groups of cases were 0-20, 21-30, 31-40, 41-50, 51-60, 61-70, 71-, the population of Gambia of each age group were adjusted in the following manner:

| Age group | Population of Gambia |
| --- | --- |
| 0-20 | $P\left( 0,19 \right)+0.2\times P(20,24)$ |
| 21-30 | $0.8\times P\left( 20,24 \right)+P\left( 25,29 \right)+0.2\times P(30,34)$ |
| 31-40 | $0.8\times P\left( 30,34 \right)+P\left( 35,39 \right)+0.2\times P(40,44)$ |
| 41-50 | $0.8\times P\left( 40,44 \right)+P\left( 45,49 \right)+0.2\times P(50,54)$ |
| 51-59 | $0.8\times P\left( 50,54 \right)+P\left( 55,59 \right)$ |
| 60 | $0.2\times P(60,64)$ |
| 61-70 | $0.8\times P\left( 60,64 \right)+P\left( 65,69 \right)+0.2\times P(70,74)$ |
| 71- | $0.8\times P\left( 70,74 \right)+P\left( 75,\infty\right)$ |

where P$\left( a,b \right)$is the number of populations among age group $a-b$

Standard population followed the same method as above.

- 1. Greece: The age groups of cases and deaths were 0-17, 18-39, 40-64, 65-. As the population of each age was available at Eurostat, it was used but after multiplying the ratio of population from United Nations and population from Eurostat for the consistency with other countries. The standard population was also adjusted in the following manner:

| Age group | Population | Standard population |
| --- | --- | --- |
| 0-17 | $\frac{P_{UN}}{P_{Eurostat}}\times P\left( 0,17 \right)$ | $Sp\left( 0,14 \right)+0.6\times Sp(15,19)$ |
| 18-39 | $\frac{P_{UN}}{P_{Eurostat}}\times P\left( 18,39 \right)$ | $0.4\times Sp\left( 15,19 \right)+Sp\left( 20,39 \right)$ |
| 40-59 | $\frac{P_{UN}}{P_{Eurostat}}\times P\left( 40,59 \right)$ | $Sp\left( 40,59 \right)$ |
| 60-64 | $\frac{P_{UN}}{P_{Eurostat}}\times P\left( 60,64 \right)$ | $Sp\left( 60,64 \right)$ |
| 65- | $\frac{P_{UN}}{P_{Eurostat}}\times P\left( 65,\infty\right)$ | $Sp(65,\infty)$ |

where P$\left( a,b \right)$is the number of populations among age group $a-b$ in Greece according to Eurostat, $P_{UN}$is the number of total populations in Greece according to United Nations Population Division’s ‘World Population Prospects: The 2019 Revision.', $P_{Eurostat}$ is the number of total populations in Greece according to Eurostat, and $Sp(a,b)$ is the number of standard populations among age group $a-b$.

- 1. Poland: The age groups of cases and deaths were 0-10, 11-20, 21-30, 31-40, 41-50, 51-60, 61-70, 71-80, 81-. As the population of each age was available at Eurostat, it was used but after multiplying the ratio of population from United Nations and population from Eurostat for the consistency with other countries. The standard population was also adjusted in the following manner:

| Age group | Population | Standard population |
| --- | --- | --- |
| 0-10 | $\frac{P_{UN}}{P_{Eurostat}}\times P\left( 0,10 \right)$ | $Sp\left( 0,9 \right)+0.2\times Sp(10,14)$ |
| 11-20 | $\frac{P_{UN}}{P_{Eurostat}}\times P\left( 11,20 \right)$ | $0.8\times Sp\left( 10,14 \right)+Sp\left( 15,19 \right)+0.2\times Sp(20,24)$ |
| 21-30 | $\frac{P_{UN}}{P_{Eurostat}}\times P\left( 21,30 \right)$ | $0.8\times Sp\left( 20,24 \right)+Sp\left( 25,29 \right)+0.2\times Sp(30,34)$ |
| 31-40 | $\frac{P_{UN}}{P_{Eurostat}}\times P\left( 31,40 \right)$ | $0.8\times Sp\left( 30,34 \right)+Sp\left( 35,39 \right)+0.2\times Sp(40,44)$ |
| 41-50 | $\frac{P_{UN}}{P_{Eurostat}}\times P\left( 41,50 \right)$ | $0.8\times Sp\left( 40,44 \right)+Sp\left( 45,49 \right)+0.2\times Sp(50,54)$ |
| 51-59 | $\frac{P_{UN}}{P_{Eurostat}}\times P\left( 51,59 \right)$ | $0.8\times Sp\left( 50,54 \right)+Sp\left( 55,59 \right)$ |
| 60 | $\frac{P_{UN}}{P_{Eurostat}}\times P\left( 60,60 \right)$ | $0.2\times Sp(60,64)$ |
| 61-70 | $\frac{P_{UN}}{P_{Eurostat}}\times P\left( 61,70 \right)$ | $0.8\times Sp\left( 60,64 \right)+Sp\left( 65,69 \right)+0.2\times Sp(70,74)$ |
| 71-80 | $\frac{P_{UN}}{P_{Eurostat}}\times P\left( 71,80 \right)$ | $0.8\times Sp\left( 70,74 \right)+Sp\left( 75,79 \right)+0.2\times Sp(80,84)$ |
| 81- | $\frac{P_{UN}}{P_{Eurostat}}\times P\left( 81,\infty\right)$ | $0.8\times Sp\left( 80,84 \right)+Sp\left( 85,\infty\right)$ |

where P$\left( a,b \right)$is the number of populations among age group $a-b$ in Poland according to Eurostat, $P_{UN}$is the number of total populations in Poland according to United Nations Population Division’s ‘World Population Prospects: The 2019 Revision.', $P_{Eurostat}$ is the number of total populations in Poland according to Eurostat, and $Sp(a,b)$ is the number of standard populations among age group $a-b$.

- 1. Indonesia: As the age groups of cases and deaths were 0-5, 6-17, 18-30, 31-45, 46-59, 60-, the similar way to Greece was used with exceptions for using United Nations Population Division’s Demographic Statistics Database (in 2010) instead of Eurostat.

| Age group | Population | Standard population |
| --- | --- | --- |
| 0-5 | $\frac{P_{2020}}{P_{2010}}\times P\left( 0,5 \right)$ | $Sp\left( 0,4 \right)+0.2\times Sp(5,9)$ |
| 6-17 | $\frac{P_{2020}}{P_{2010}}\times P\left( 6,17 \right)$ | $0.8\times Sp\left( 5,9 \right)+Sp\left( 10,14 \right)+0.6\times Sp\left( 15,19 \right)$ |
| 18-30 | $\frac{P_{2020}}{P_{2010}}\times P\left( 18,30 \right)$ | $0.4\times Sp\left( 15,19 \right)+Sp\left( 20,29 \right)+0.2\times Sp(30,34)$ |
| 31-45 | $\frac{P_{2020}}{P_{2010}}\times P\left( 31,45 \right)$ | $0.8\times Sp\left( 30,34 \right)+Sp\left( 35,44 \right)+0.2\times Sp\left( 45,49 \right)$ |
| 46-59 | $\frac{P_{2020}}{P_{2010}}\times P\left( 46,59 \right)$ | $0.8\times Sp\left( 45,49 \right)+Sp\left( 50,59 \right)$ |
| 60- | $\frac{P_{2020}}{P_{2010}}\times P\left( 60,\infty\right)$ | $Sp(60,\infty)$ |

where P$\left( a,b \right)$is the number of populations among age group $a-b$ in Indonesia according to United Nations Population Division’s Demographic Statistics Database (in 2010), $P_{2010}$is the number of total populations in Indonesia according to United Nations Population Division’s Demographic Statistics Database, $P_{2020}$ is the number of total populations in Indonesia according to United Nations Population Division’s ‘World Population Prospects: The 2019 Revision.', and $Sp(a,b)$ is the number of standard populations among age group $a-b$.

- 1. USA: As the age groups of cases and deaths were .0-4, 5-17, 18-29, 30-39, 40-49, 50-64, 65-74, 75-84, 85-, the similar way to Greece was used with exceptions for using United States Census Bureau instead of Eurostat.

| 1. Age group | Population | Standard population |
| --- | --- | --- |
| 0-4 | $\frac{P_{UN}}{P_{Census}}\times P\left( 0,4 \right)$ | $Sp\left( 0,4 \right)$ |
| 5-17 | $\frac{P_{UN}}{P_{Census}}\times P\left( 5,17 \right)$ | $Sp\left( 5,14 \right)+0.6\times Sp\left( 15,19 \right)$ |
| 18-29 | $\frac{P_{UN}}{P_{Census}}\times P\left( 18,29 \right)$ | $0.4\times Sp\left( 15,19 \right)+Sp\left( 20,29 \right)$ |
| 30-39 | $\frac{P_{UN}}{P_{Census}}\times P\left( 30,39 \right)$ | $Sp\left( 30,39 \right)$ |
| 40-49 | $\frac{P_{UN}}{P_{Census}}\times P\left( 40,49 \right)$ | $Sp(40,49)$ |
| 50-59 | $\frac{P_{UN}}{P_{Census}}\times P\left( 50,59 \right)$ | $Sp\left( 50,59 \right)$ |
| 60-64 | $\frac{P_{UN}}{P_{Census}}\times P\left( 60,64 \right)$ | $Sp\left( 60,64 \right)$ |
| 65-74 | $\frac{P_{UN}}{P_{Census}}\times P\left( 65,74 \right)$ | $Sp\left( 65,74 \right)$ |
| 75-84 | $\frac{P_{UN}}{P_{Census}}\times P\left( 75,84 \right)$ | $Sp\left( 75,84 \right)$ |
| 85- | $\frac{P_{UN}}{P_{Census}}\times P\left( 85,\infty\right)$ | $Sp(85,\infty)$ |

where P$\left( a,b \right)$is the number of populations among age group $a-b$ in USA according to United States Census Bureau, $P_{UN}$is the number of total populations in USA according to United Nations Population Division’s ‘World Population Prospects: The 2019 Revision.', $P_{Census}$ is the number of total populations in USA according to United States Census Bureau, and $Sp(a,b)$ is the number of standard populations among age group $a-b$.

1. UK: In the process of unification, the population structures of each nation (England, Scotland, Wales and Northern Ireland) were utilized from Office for National Statistics.
