# Supplementary material for: The age-standardized incidence, mortality, and case fatality rates of COVID-19 in 79 countries: a cross-sectional comparison and their correlations with associated factors
Source: Epidemiol Health. 2021 Sep 8;43:e2021061. doi: 10.4178/epih.e2021061 (PMC8611321; doi:10.4178/epih.e2021061)
Supplement: Supplementary Material 3. — Pearson's correlation coefficients and partial correlation coefficients among the log-transformed variables as of October 8, 2020. (n=55) [file epih-43-e2021061-suppl3.docx]

Supplementary Material 3. Pearson's correlation coefficients and partial correlation coefficients among the log-transformed variables as of October 8, 2020. (n=55)

| Variables | All ages | | | | | |  | Age≥60 | | | | | |
| --- | --- | --- | --- | --- | --- | --- | --- | --- | --- | --- | --- | --- | --- |
|  | Incidence | p-value | Mortality | p-value | CFR | p-value |  | Incidence | p-value | Mortality | p-value | CFR | p-value |
| GDP per capita | 0.24 | 0.0851 | 0.06 | 0.6976 | -0.43 | 0.0033 |  | 0.05 | 0.7029 | 0.13 | 0.4025 | -0.14 | 0.3796 |
| No. of beds | 0.09 | 0.5165 | -0.15 | 0.3303 | -0.47 | 0.0012 |  | -0.02 | 0.8603 | -0.1 | 0.5259 | -0.29 | 0.0527 |
| No. of Doctor | 0.35 | 0.0086 | 0.14 | 0.3700 | -0.43 | 0.0036 |  | 0.2 | 0.1451 | 0.2 | 0.1935 | -0.15 | 0.3246 |

*All variables were log-transformed.
